# Development of the Norwegian diet index and the Norwegian lifestyle index and evaluation in a national survey

**DOI:** 10.29219/fnr.v67.9217

**Published:** 2023-09-29

**Authors:** Hege Berg Henriksen, Hedda Beate Berg, Lene Frost Andersen, Harald Weedon-Fekjær, Rune Blomhoff

**Affiliations:** 1 Department of Nutrition, Institute of Basic Medical Sciences, Faculty of Medicine, University of Oslo, Oslo, Norway; 2 Institute of Basic Medical Sciences, Faculty of Medicine, University of Oslo, Oslo, Norway; 3 Oslo Center for Biostatistics and Epidemiology, Research Support Services, Oslo University Hospital, Oslo, Norway; 4 Division of Cancer Medicine, Oslo University Hospital, Oslo, Norway

**Keywords:** diet index, lifestyle index, food-based dietary guidelines, national lifestyle recommendations, body weight, physical activity, alcohol, tobacco

## Abstract

**Background:**

Dietary and lifestyle indices are composite tools that are used to estimate risk of health outcomes.

**Objective:**

We aimed to develop a diet and a lifestyle index assessing adherence to the national guidelines in Norway, and to investigate adherence in a nationwide survey of healthy subjects (Norkost3).

**Design:**

Cut-off values for the indices were based on the Norwegian food based dietary guidelines and national lifestyle guidelines. Adherence was evaluated in the Norkost3 (*n* = 1,787).

**Results:**

Twelve dietary components were included in the diet index 1) fruit and berries, 2) vegetables, 3) whole grains, 4) unsalted nuts, 5) fish, 6) low-fat dairy products, 7) margarine/oils, 8) red meat, 9) processed meat, 10) foods rich in sugar and fat, 11) drinks with added sugar, and 12) dietary supplements. Each of the components was assigned a value of 0, 0.5 or 1 corresponding to low, intermediate and high adherence, except for plant-based foods, which were assigned a value of 0, 1.5 or 3, providing a composite diet index ranging from 0 to 20 points. The five components in the lifestyle index (i.e. diet, body mass index (BMI), physical activity, tobacco and alcohol) was assigned a value of 0, 0.5 or 1, giving a final score ranging from zero to five points. In Norkost3, 49% (95% CI: 47, 52) of the participants had low adherence to the diet component, whereas only 2% (95% CI: 2, 3) achieved high adherence, although most of the subjects had high educational level. High adherence to the recommendations of BMI, tobacco and alcohol intake was observed in 50% (95% CI: 47, 52), 72% (95% CI: 70, 74) and 68% (95% CI: 66, 70) of the participants, respectively. Due to the lack of data on physical activity, adherence to this component in the lifestyle index is not presented in this study.

**Conclusion:**

The new diet and lifestyle indices assess adherence to the Norwegian food-based dietary guidelines (FBDGs) and other national lifestyle guidelines. In this study, half of the subjects had low diet and lifestyle index scores. There is a need to implement interventions to improve this by focusing on the specific lifestyle components with low adherence.

## Popular scientific summary

- Dietary and lifestyle indices are composite tools that are used to estimate risk of health outcomes.
- We developed a diet and a lifestyle index assessing adherence to the national guidelines in Norway, and investigated adherence in a nationwide survey of healthy subjects (Norkost3).
- Half of the subjects in Norkost3 had low diet and lifestyle index scores.
- Interventions focusing on the specific lifestyle components with low adherence in a population are needed.

Evidence linking dietary patterns with different health outcomes has accumulated over the past two decades. Dietary patterns describe the combination of foods that a person eats including the amount and/or frequency (1). In addition to the more traditional investigation of single foods or nutrients, dietary patterns may also be more predictive of disease risk due to the variety of foods with complex combinations of nutrients, other bioactive compounds and food-matrix (2). Thus, dietary patterns provide a more comprehensive understanding of the relationship between overall food consumption and disease outcomes.

A variety of established methods exist to derive dietary patterns, where index-based patterns (or diet index) and exploratory patterns represent the two most common approaches (1). The former, also known as *a priori* approach, is based on already established knowledge (1, 3). The result achieved using an index-based pattern indicate how well a person comply with a certain cultural-based and accepted healthy dietary pattern, for example the Mediterranean diet (4) and the new Nordic Food Index (5) or food-based dietary guidelines (FBDGs) such as the dietary guidelines for Americans (the Healthy Eating Index [HEI]) (6, 7). The latter method, on the contrary, is an *a posteriori* approach based on statistical analysis of current data, such as principal component analysis, cluster and factor analysis, and reduced ranked regression (1, 3).

Often, additional lifestyle factors are included in the index such as physical activity, weight status, alcohol and smoking. These indices are termed lifestyle indices. One example of a lifestyle index is the World Cancer Research Fund (WCRF) score, which is based on the cancer prevention recommendations published by the WCRF/American Institute of Cancer Research (8).

To date, no indices are available that are based on the Norwegian FBDG (9) and national guidelines for other lifestyle factors (10). The aim of the present study was to develop a diet index and a lifestyle index that are based on the Norwegian FBDG and national lifestyle guidelines in Norway. Furthermore, we also aimed to investigate how an adult Norwegian population adhere to these new indices.

## Methods

### Development of the Norwegian diet index

The components included in the diet index are based on the recommendations in the Norwegian FBDG (9). The recommendations include both beneficial components (i.e. foods that should be eaten) and disadvantageous components (i.e. that should be minimized or avoided). The operationalization of the main recommendations in Norwegian FBDGs that has been described previously (11, 12), was used in the construction of the diet index. In short, the Norwegian FBDG consist of both quantitative and qualitative recommendations of prepared foods (i.e. ready to eat). The quantitative recommendations were used as the limit of high adherence to the corresponding components in the diet index (Table 1). A whole grain factor developed in Henriksen et al. was used to estimate whole grain intake from whole grain products (Table 1) (12). Weekly recommendations were translated into daily intakes and by using the lower range of recommended intake. For example, recommended intake of fish, including both lean and fatty fish is 300–450 g per week (of which 200 g should be fatty fish), was translated into the lower range of recommended intake of 300 g per week or 43 g/d. This amount refers to prepared lean and fatty fish ready to eat.

**Table 1:** An overview of the components included in the Norwegian diet index with cut-off values and scoring

The Norwegian diet index				
Component number	Diet component	Quantitative recommendation	Level of adherence	Maximal points
1.	Fruit and berries^a^	≥250 g/d	**3**: ≥250; **1.5**: 125–250; **0**: <125	**3**
2.	Vegetables^b^	≥250 g/d	**3**: ≥250; **1.5**: 125–250; **0**: <125	**3**
3.	Whole grains^c^	**Women:** ≥70 g/d	**Women: 3**: ≥70; **1.5**: 35–70; **0**: <35	**3**
		**Men:** ≥90 g/d	**Men: 3:** ≥90; **1.5**: 45–90; **0**: <45	
4.	Unsalted nuts	**BMI <25:** ≥20 g/d	**BMI <25: 3:** ≥20; **1.5:** 10–20; **0:** <10	**3**
		**BMI ≥25:** 20 g/d ≤ nuts <30 g/d	**BMI ≥25: 3:** 20 ≤ nuts <30; **1.5:** 10–20; **0:** <10 OR > 30	
5.	Fish (lean and fatty fish)	≥43 g/d	**1:** ≥43; **0.5**: 21.5–43; **0**: <21.5	**1**
6.	Low-fat dairy products^d^	≥100 g/d	**1:** ≥100; **0.5:** 50–100; **0:** <50	**1**
7.	Margarine/oils	Preferable choose oils and margarine high in unsaturated FAs and low in saturated FAs	**1**: soft margarine/oils; **0.5**: soft margarine/oils and hard butter; **0**: hard butter	**1**
8.	Red meat^e^	≤71 g/d	**1:** <35.5; **0.5:** 35.5–71; **0:** ≥71	**1**
9.	Processed meat^f^	≤20 g/d	**1:** <10; **0.5**: 10–20; **0:** >20	**1**
10.	Foods rich in sugars and fat^g^	≤20 g/d	**1:** <10; **0.5**: 10–20; **0:** >20	**1**
11.	Beverages with added sugar, such as carbonated drinks	≤20 g/d	**1:** <10; **0.5**: 10–20; **0:** >20	**1**
12.	Dietary supplements^h^	0 unit/d	**1:** = 0; **0**: >0	**1**
**Total diet index score**				**20**

FA: fatty acid.

a Include maximum one glass (200 g) of juice as one portion of fruit (100 g), not jam.

b Not included legumes or potatoes.

c Intake of whole grains was calculated using a whole grain factor (with the assumption that bread contains 60% flour and boiled rice/pasta contains 30% cereal):

Bread with 0–25% wholemeal flour: (60*0)/10,000 = 0

Bread with 25–50% wholemeal flour: (60*25)/10,000 = 0.15

Bread with 50–75% wholemeal flour: (60*50)/10,000 = 0.30

Bread with 75–100% wholemeal flour: (60*75)/10,000 = 0.45

Whole grain crisp bread = 1

Sweetened cereals = 0.25

Unsweetened cereals = 0.75

Brown rice = 0.30

Whole grain pasta = 0.30

d Includes lean milk with less than 1.5% fat, dairy products (not cheese and milk) containing more than 20% fat and/or energy content more than 950–1,150 kJ and cheese containing less than 17% fat, cheese labelled with light/fat reduced or containing energy less than 950–1,150 kJ.

e Includes non-processed and processed beef, pork, lamb and goat.

f Includes processed all forms of processed meat.

g Includes cakes, dessert, ice-cream, candy and snacks.

h It is not recommended to have an intake of dietary supplementation, but eat fresh vegetables, fruits, low-fat dairy products, whole grains, fish (lean and fatty fish) and lean meat in order to reach the recommended daily intake of nutrients. Therefore, intake of dietary supplements is given 0 point in the score. However, if a person is advised by the physician to use dietary supplements, he/she should continue to follow this recommendation.

The qualitative recommendations from the Norwegian FBDG included in the diet index were translated into quantitative recommendation following similar procedure as in Henriksen et al. (12). Recommendations regarding foods that should be limited or reduced were operationalized into quantitative limits as described previously (12). For example, the recommendation to limit consumption of drinks with added sugar was translated into not more than 20 g/d. For recommendations of foods that should be consumed daily, *a priori* defined minimum amount was made. For example, a daily intake of low-fat dairy products was defined as at least half a portion of low-fat dairy products per day (i.e. ≥ 100 g/d) (Table 1) (12).

### Development of the Norwegian lifestyle index

The diet index was included in the lifestyle index as one of the total five components. The other four components were lifestyle behaviors (i.e. BMI, physical activity, use of tobacco [e.g. smoking and snuff] and alcohol) based on the national lifestyle recommendations. All of these components are important risk factors for health outcomes (1, 9, 13–17).

### The Norwegian national dietary survey (Norkost3)

This study included data from Norkost3, which is the third national dietary survey in Norway conducted between 2010 and 2011 (18). The study has been described in detail elsewhere (18). Data were collected from adults aged between 18 and 70 years using a 24-h recall conducted twice with 4 weeks between the first and the second interview. Intakes of foods were recorded as consumed and coded in the food composition database and food and nutrient calculation system KBS, at the Department of Nutrition, University of Oslo. Standardized portion sizes and household measurements as defined in KBS were used when estimating amounts of foods (18).

## Results

### The Norwegian diet index and the Norwegian lifestyle index

All components, cut-off values, and operationalization of recommendations included in both indices are presented in Tables 1 and 2. The 12 components included in the diet index were i) fruit and berries, ii) vegetables, iii) whole grains, iv) unsalted nuts, v) fish (i.e. lean and fatty fish), vi) low-fat dairy products, vii) margarine/oils, viii) red meat (i.e. unprocessed and processed red meat), ix) processed meat (i.e. all forms of processed meat), x) foods rich in sugar and fat, xi) drinks with added sugar, and xii) dietary supplements. We selected a three-level scoring approach in which the three categories represent low adherence, intermediate adherence, and high adherence. We used the cut-off values for full adherence as defined in Henriksen et al. for all components included in the diet index (Table 1) (12). Cut-off values for low and intermediate adherence were set by taking the value needed to fulfill the recommendation and divide into three groups with approximately similar ranges in scores. For instance, 250 g of fruits and berries per day are needed to fulfill the recommendation. Thus, the cut-off values for low, intermediate and high adherence were set to <125 g, 125–250 g and ≥250 g, respectively (Table 1). We selected a dichotomous score for one of the 12 components, that is use (=0) or not use (=1) of dietary supplements. Sex-specific cut-off values were developed for the whole grains component reflecting different advice for sexes (Table 1). Eight out of the twelve components were assigned a value of 0, 0.5 or 1 based on adherence. Since the main message of the Norwegian FBDG (9) is to eat a diet consisting of mainly plant-based foods, these components (i.e. fruit and berries, vegetables, unsalted nuts and whole grains) were assigned 60% of the total diet score (i.e. maximum 12 out of 20 points). Therefore, all plant-based components were assigned a value of 0, 1.5 or 3.

**Table 2:** The Norwegian lifestyle index with cut-off values and scoring

Component number	Lifestyle component	Quantitative recommendation	Level of adherence	Maximal points
1.	Diet	Total score: 0–20	**1**: 14–20; **0.5**: 8–13; **0**: 0–7	**1**
2.	Body weight	Healthy weight (18.5–24.9 kg/m^2^)	**1**: 18.5–24.9; **0.5:** 25–29.9; **0:** <18.5 OR ≥ 30	**1**
3.	Physical activity	150 min/week of MVPA	**1:** ≥150; **0.5**: 75–149.9; **0**: <75	**1**
4.	Tobacco^a^	0 (i.e. no user)	**1:** =0; **0**: >0	**1**
5.	Alcohol^b^	0 g/d	**1:** 0; **0.5:** >0–4.29; **0**: >4.29	**1**
**Total lifestyle index score**				**5**

MVPA: moderate-to-vigorous physical activity.

a Includes smoking and snuff.

b Alcohol intake was estimated using an alcohol factor, which was developed based on the amount of alcohol (per 100 g of edible food) in a standard unit of beer, wine, and spirits.

Beer with 4.7 vol-% alcohol: 3.8/100 = 0.038 (beer factor)

Wine with 10.2 vol-% alcohol: 10.2/100 = 0.102 (wine factor)

Spirits with 40 vol-% alcohol: 33.7/100 = 0.337 (spirits factor)

Overall, the sum of each component in the diet index represents the final score (ranging between 0 and 20 points), reflecting how well individuals comply with the Norwegian FBDGs for a healthy dietary pattern (Table 1).

The Norwegian lifestyle index consisted of five components including diet, BMI, physical activity, tobacco, and alcohol (Table 2). The three-level scoring approach was also applied in this index for all components, with low-, intermediate-, and high-adherence, except for tobacco, which had an dichotomous scoring approach for use (i.e. 0 points) or not use (i.e. 1 point). The diet component was based on the Norwegian diet index and cut-off criteria were defined as the division of the total score of 20 points into three groups with approximately similar ranges in scores (i.e. low [0: 0–7 points], intermediate [0.5: 8–13 points] and high [1: 14–20 points]). A maximum of one point of each of the five components could be allotted, resulting in a total score ranging from zero to five points (Table 2).

We developed an alcohol factor used to estimate the amount of alcohol in different alcoholic beverages, such as beer, wine and spirits (Table 2). It is recommended to be physical active in moderate to vigorous intensity in at least 150 min per week (15, 16), and the scores of adherences were divided into three levels with low (0: <75 min), intermediate (0.5: 75–149.9 min) and high (1: ≥150 min) adherence. A healthy weight was defined as having a BMI within 18.5–24.9 kg/m^2^ (9) and were also divided into three levels of adherence as shown in Table 2. Use of tobacco (i.e. smoking and snuff) is not recommended and therefore level of adherence was divided into two levels (i.e. user vs. no user) (Table 2) (10).

### Investigating adherence to the Norwegian diet index and Norwegian lifestyle index in the Norkost3 survey

#### Participants’ characteristics

Participants’ characteristics for the total study population and stratified by sex are shown in Table 3. Data were available for 1,787 subjects, of which 48% were men and 52% were women. Mean (±SD) age and BMI were 46 (14) years and 25 (4) kg/m^2^, respectively. Most of the participants were classified as normal weight (50%) followed by overweight (37%). Twenty-one percent and 10% used smoke and snuff, respectively. The majority of the respondents had higher education from either college or university (53%). Mean dietary intake of energy and food groups included in the Norwegian diet index are presented in Supplementary Table 1.

**Table 3:** Characteristics of the total study population and stratified by sex in Norkost3

Variable	*N*	Total (*n* = 1,787)	Men (*n* = 862)	Women (*n* = 925)
**Age (yrs.), mean (±SD)**	1,787	46.0 (13.9)	46.9 (14.4)	45.2 (13.4)
**Age groups, % (** *n* **) (years)**	1,787			
18–29		16 (281)	16 (138)	16 (143)
30–39		17 (305)	16 (136)	18 (169)
40–49		24 (435)	21 (179)	28 (256)
50–59		22 (385)	22 (192)	21 (193)
60–71		21 (381)	25 (217)	18 (164)
**Anthropometry**				
Height (cm), mean (±SD)	1,786	174.1 (9.0)	181.1 (6.2)	167.6 (5.9)
Weight (kg), mean (±SD)	1,757	77.5 (15.4)	86.2 (13.2)	69.2 (12.6)
BMI (kg/m^2^), mean (±SD)	1,756	25.4 (4.0)	26.3 (3.5)	24.6 (4.2)
**BMI classification, % (** *n* **)** ^a^	1,756			
Underweight		1 (22)	0 (2)	2 (20)
Normal weight		50 (870)	40 (344)	59 (526)
Overweight		37 (652)	46 (394)	29 (258)
Obese		12 (212)	14 (121)	10 (91)
**Smoking status, % (** *n* **)**	1,787			
Yes		21 (377)	20 (176)	22 (201)
No		79 (1,410)	80 (686)	78 (724)
**Snuff status, % (** *n* **)**	1,787			
Yes		10 (170)	16 (140)	3 (30)
No		91 (1,617)	84 (722)	97 (895)
**Educational level, % (** *n* **)**	1,784			
Primary school		5 (94)	6 (55)	4 (39)
Secondary school		42 (752)	44 (377)	41 (375)
College/university		53 (938)	50 (429)	55 (509)
**Family status, % (** *n* **)**	1,787			
Living alone		17 (301)	18 (151)	16 (150)
Family without children		33 (596)	36 (314)	31 (282)
Family with children		36 (651)	34 (296)	38 (355)
Other		13 (239)	12 (101)	15 (138)

Abbreviations: *n*, number; SD, standard deviation;; BMI, body mass index.

a underweight: <18.5 kg/m^2^, normal weight: 18.5–24.9 kg/m^2^, overweight: 25–29.9 kg/m^2^, obese: ≥30 kg/m^2^.

#### Adherence to the Norwegian diet index and the Norwegian lifestyle index

**Fig. 1:**
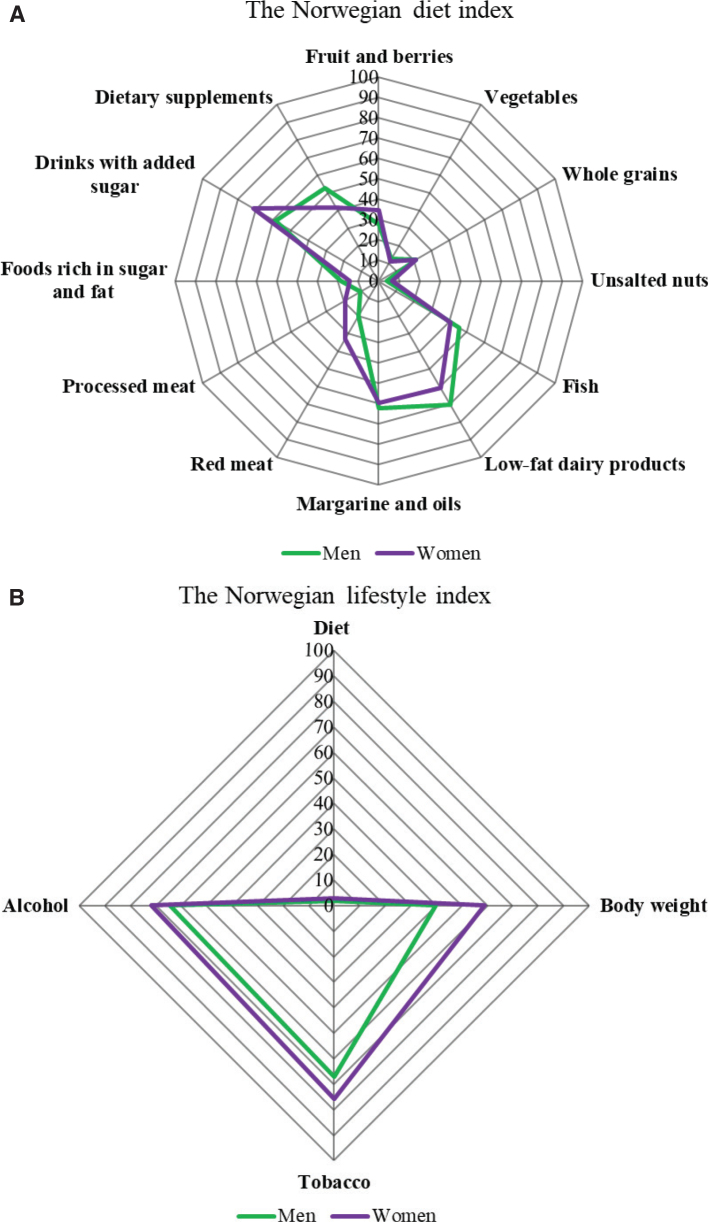
Adherence with dietary and lifestyle recommendations for men (green) and women (purple). Data available for *n* = 1,787 except for nuts (*n* = 1,786) and body weight (*n* = 1,756). Each axis represents one index component that ranges from 0% (no adherence) to 100% (high adherence). (A) Proportion (%) that is highly adherent to the individual components of the Norwegian diet index. (B) Proportion (%) that is highly adherent to the individual components of the Norwegian lifestyle index.

Table 4 presents the level of adherence (i.e. low, intermediate, and high adherence) to the dietary recommendations in the total study population and in both sexes separately. The percentage of participants with high adherence with each of the 12 recommendations in the diet index ranged from 5 to 65%. Women were more adherent to recommendations about fruit and berries, unsalted nuts, red and processed meat, and drinks with added sugar compared with men. On the contrary, men were more adherent to low-fat dairy products and supplements than women. Intakes of fish and meat consumption were slightly overestimated due to the inclusion of raw or not prepared food items in the calculation of intakes from the Norwegian Food Composition Table (KBS). Data on physical activity collected in Norkost3 was not suitable for this study. Therefore, we only tested the adherence with four of the five lifestyle recommendations (Table 5). The proportion of individuals with high adherence to each of the four recommendations (i.e. diet, BMI, tobacco and alcohol) ranged from 2 to 72%. Women were more adherent to body weight, tobacco, and alcohol. No difference was seen in the overall adherence to diet. The adherence to dietary and lifestyle recommendations is depicted in Fig. 1A and B, respectively.

**Table 4:** Adherence to dietary recommendations in the total study population and stratified by sex, with 95% based on Jeffreys interval for binominal proportions

Index component	Cut-off points	Level of adherence^a^	Percent adherence with 95% confidence interval		
			Total (*n* = 1,787)	Men (*n* = 862)	Women (*n* = 925)
Fruit and berries	≥250 g/d	High adherence	31 (29, 34)	28 (25, 31)	35 (32, 38)
		Intermediate adherence	30 (28, 32)	28 (25, 31)	32 (29, 35)
		Low adherence	39 (37, 41)	45 (41, 48)	33 (30, 36)
Vegetables	≥250 g/d	High adherence	12 (10, 13)	13 (10, 15)	11 (9, 13)
		Intermediate adherence	38 (36, 41)	35 (31, 38)	42 (39, 45)
		Low adherence	50 (48, 52)	53 (50, 56)	47 (44, 50)
Whole grains	Women: ≥70 g/d	High adherence	21 (19, 23)	21 (19, 24)	21 (18, 24)
	Men: ≥90 g/d	Intermediate adherence	38 (35, 40)	36 (33, 39)	39 (36, 42)
		Low adherence	41 (39, 44)	43 (39, 46)	40 (37, 43)
Unsalted nuts^b^	BMI<25: ≥20 g/d	High adherence	5 (4, 6)	4 (3, 5)	7 (5, 8)
	BMI≥25: 20 g/d	Intermediate adherence	5 (4, 6)	4 (3, 5)	6 (4, 7)
	≤ nuts <30 g/d	Low adherence	90 (89, 91)	93 (91, 94)	88 (85, 90)
Fish	≥43 g/d	High adherence	43 (41, 45)	46 (42, 49)	41 (38, 44)
		Intermediate adherence	8 (7, 10)	8 (6, 10)	8 (7, 10)
		Low adherence	49 (46, 51)	46 (43, 50)	51 (48, 54)
Low-fat dairy products	≥100 g/d	High adherence	65 (63, 67)	70 (67, 73)	61 (57, 64)
		Intermediate adherence	8 (7, 10)	5 (4, 7)	11 (9, 13)
		Low adherence	27 (25, 29)	25 (22, 28)	28 (26, 31)
Margarine/oils	Oils and margarine	High adherence	61 (59, 63)	63 (59, 66)	60 (57, 63)
		Intermediate adherence	22 (21, 24)	22 (19, 25)	23 (20, 26)
		Low adherence	17 (15, 18)	16 (13, 18)	18 (15, 20)
Red meat	≤71 g/d	High adherence	27 (25, 29)	20 (17, 23)	33 (30, 36)
		Intermediate adherence	17 (15, 19)	15 (12, 17)	19 (16, 21)
		Low adherence	57 (54, 59)	66 (62, 69)	48 (45, 52)
Processed meat	≤20 g/d	High adherence	15 (13, 16)	10 (8, 13)	19 (16, 21)
		Intermediate adherence	10 (9, 12)	9 (8, 12)	11 (9, 13)
		Low adherence	75 (73, 77)	80 (78, 83)	70 (67, 73)
Foods rich in sugars and fat	≤20 g/d	High adherence	16 (15, 18)	19 (16, 21)	14 (12, 17)
		Intermediate adherence	7 (6, 9)	7 (5, 8)	8 (7, 10)
		Low adherence	76 (74, 78)	75 (72, 78)	78 (75, 80)
Drinks with added sugar	≤20 g/d	High adherence	65 (63, 67)	59 (55, 62)	71 (68, 74)
		Intermediate adherence	0 (0, 1)	0 (0, 1)	1 (0, 1)
		Low adherence	35 (32, 37)	41 (38, 44)	28 (26, 31)
Dietary supplements	0 unit/d	High adherence	47 (45, 49)	53 (49, 56)	42 (39, 45)
		Low adherence	53 (51, 55)	47 (44, 51)	58 (55, 62)

a Level of adherence as defined in Table 1.

b *n* = 1,786.

**Table 5:** Adherence to lifestyle recommendations in the total study population and stratified by sex, with 95% based on Jeffreys interval for binominal proportions

Index component	Cut-off points	Level of adherence^a^	Percent adherence with 95% confidence interval		
			Total (*n* = 1,787)	Men (*n* = 862)	Women (*n* = 925)
Diet	Total score: 0–20	High adherence	2 (2, 3)	2 (1, 3)	3 (2, 4)
		Intermediate adherence	48 (46, 51)	44 (40, 47)	53 (50, 56)
		Low adherence	49 (47, 52)	55 (51, 58)	44 (41, 47)
Body weight^b^	Healthy weight (18.5–24.9 kg/m^2^)	High adherence	50 (47, 52)	40 (37, 43)	59 (56, 62)
		Intermediate adherence	37 (35, 39)	46 (43, 49)	29 (26, 32)
		Low adherence	13 (12, 15)	14 (12, 17)	12 (10, 15)
Tobacco (smoking and snuff)	None user	High adherence	72 (70, 74)	67 (64, 70)	76 (73, 79)
		No adherence	28 (26, 30)	33 (30, 36)	24 (21, 27)
Alcohol	0 g/d	High adherence	68 (66, 70)	64 (61, 68)	72 (69, 75)
		Intermediate adherence	1 (0, 1)	1 (0, 1)	1 (0, 1)
		Low adherence	31 (29, 33)	35 (32, 38)	28 (25, 31)

a Level of adherence as defined in Table 2.

b *n* = 1,756.

## Sensitivity of the components in the Norwegian diet index

**Fig. 2:**
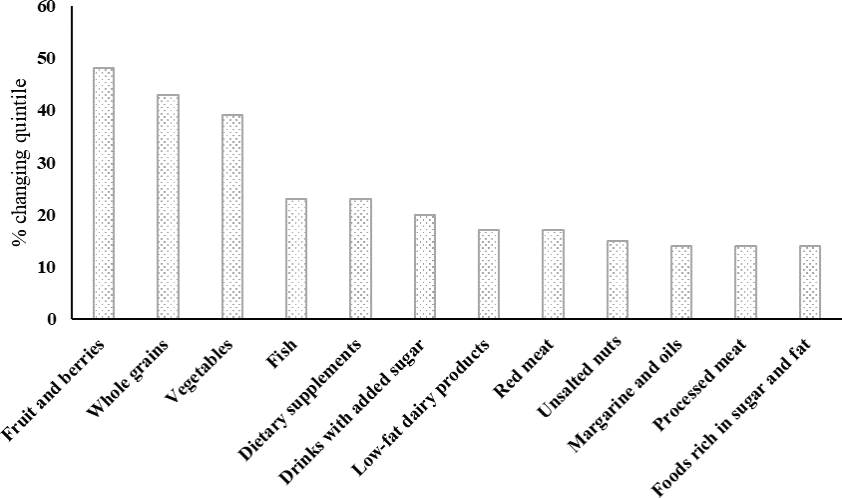
Percentage of participants who change quintile after excluding each individual component of the Norwegian diet index. Data available for *n* = 1,787 except for nuts (*n* = 1,786).

Figure 2 illustrates the percentage of participants changing quintile when excluding a specific index component. Fruit and berries, whole grains, and vegetables contributed the most to the total index score. As evident from the figure, 48, 43, and 39% of the participants changed quintile when removing fruit and berries, whole grains, and vegetables, respectively.

The least contributing components were foods rich in sugar and fat, processed meat, margarines and oils in which 14% participants changed quintile when one of them was removed.

## Discussion

This study describes the development of two indices: a diet index and a lifestyle index. Both indices are based on the national diet and lifestyle recommendations in Norway. The diet index is composed of the main recommendations in the Norwegian FBDG. In addition, the lifestyle index also integrate BMI, physical activity, and use of tobacco and alcohol.

The purpose of indices may vary. The original HEI-index, and many of the varieties developed based on HEI, as well as the Mediterranean diet score and Diet Quality Index (DQI) aims to assess adherence to dietary guidelines with a major focus on cardiovascular diseases, while the DASH index focuses on dietary components that reduce risk of hypertension (4, 6, 7, 19–21). Additionally, the 2018 WCRF/AICR-score aims to assess adherence to risk factors for cancer (8). The Norwegian diet index, presented in this article, aims at combining all the diet risk factors identified for all major diet-related chronic diseases.

The components included in different diet and lifestyle indices varies based on the purpose of the index and their use. For example, the most recent HEI-index (HEI-2015) include the following components: total fruit, whole fruit (total fruit excluding juice), total vegetables, dark green vegetables, legumes, whole grains, dairy, total protein foods (lean fraction only), seafood, eggs, soy products, nuts and seeds, refined grains, saturated fatty acids, polyunsaturated fatty acids, monounsaturated fatty acids, sodium, calories from added sugars, and total calories (7).

The 2018 WCRF/AICR-score include both diet and other lifestyle components: i) BMI and waist circumference, ii) physical activity, iii) fruits and vegetables and fiber, iv) fast foods and other processed foods, v) red and processed meat, vi) surgery beverages, vii) alcohol and viii) breastfeeding (optional) (8).

In contrast, the new Norwegian diet index, which is broader and more food based than those discussed above, include i) fruit and berries, ii) vegetables, iii) whole grains, iv) unsalted nuts, v) fish, vi) low-fat dairy products, vii) margarine/oils, viii) red meat, xi) processed meat, x) foods rich in sugar and fat, xi) drinks with added sugar, and xii) dietary supplements. These components reflect all major aspects of the Norwegian FBDG which focus on diet risk factors for all major chronic diseases (9).

The Norwegian lifestyle index also contains the four major lifestyle risk factors physical activity, body weight, alcohol and smoking (17). The components of the Norwegian lifestyle index is neither based on one particular disease, but aims at measure adherence to the major lifestyle risk factors for all major diet-related chronic diseases (17).

The scoring of components vary in different indices (Table 6). The HEI-2015 use an almost continuous scoring of each component with values ranging from 0 to 10, the DASH score use quintiles giving 1 to 5 points, the most recent 2018 WCRF/AICR-score use the values 0, 0.5 and 1 for each component, while some others use a dichotomous scoring of components (e.g. the original WCRF/AICR-index and Nordic Food Index). Dichotomous scoring is often used when data are based on qualitative questionnaires.

**Table 6:** Components and scoring of different indices

Index	Components	Scoring	Weighting
Norwegian diet index	Fruit and berries, vegetables, whole grains, unsalted nuts, fish, low-fat dairy products, margarine/oils, red meat, processed meat, foods rich in sugar and fat, drinks with added sugar, dietary supplements	3-part (0, 0.5, 1/0,1.5, 3)	Yes
Norwegian lifestyle index	Diet, physical activity, BMI, alcohol, smoking	3-part (0, 0.5, 1)	No
HEI-2010 (6)	Vegetable and fruits, grains, dairy, protein foods, fats, sodium, alcohol, (calorie balance and physical activity, eating pattern)	5–10 part	No/yes-subcomponents
HEI-2015 (7)	Total fruits, whole fruits, total vegetables, greens and beans, whole grains, dairy, total protein foods, sea foods and plant protein, fatty acids, refined grains, sodium, added sugars, saturated fats	5–10 part	No/yes- subcomponents
WCRF-2007 (29)	Body fatness, physical activity, foods that promote weight gain, plant foods, red and processed meat, and alcohol	2-part (0, 1)	No
WCRF-2018 (8)	Body fatness, physical activity, wholegrains/fruits/vegetables/beans, fast foods/UPF, red and processed meat, sugar-sweetened drinks, alcohol, breastfeeding	3-part (0, 0.5, 1)	No
DASH-score (21)	Fruits, vegetables, nuts and legumes, whole grains, low-fat dairy products, sodium, red and processed meat, sweetened beverages	Quintiles: Q1:1 point Q5: 5 points	No
Healthy Nordic food index (5)	Fish, cabbage, whole grain rye, whole grain oats, apples/pears, root vegetables	2-part (above/below population median)	No

We selected to use a three-level scoring system for each component, which is also used in the most recent 2018 WCRF/AICR-score, since national guidelines often are a combination of qualitative and quantitative recommendations. A three-level scoring is more sensitive to changes in adherence than a dichotomous scoring and partially meeting a recommendation will likely confer some benefit. While a 5- and 10-part scoring can be even more sensitive, the operationalization of qualitative recommendations is challenging. In addition, a 5- and 10-part scoring also requires extensive registration of data, which is often not available in large surveys (7, 22).

A major recommendation in the Norwegian FBDG and the WCRF recommendations is to have a predominantly plant-based diet (9, 13). The WCRF recommends that about two thirds of your total diet should be composed of plant-based foods (13). Moreover, intakes of nuts and oils have been shown to reduce the risk of CVD in the PREDIMED study (23). A plant-based diet is also recommended by UN due to being beneficial in a climate and environmental sustainability perspective (24), also supported in the report published by the Norwegian health authorities in 2017 evaluating sustainability aspects for each of the Norwegian FBDGs (25). Accordingly, we selected to score the four plant-based components 0, 1.5 and 3 while the eight other components were scored 0, 0.5 and 1. Thus, if compliant to all plant-based food recommendations, it will contribute 12 (60%) out of maximally 20 points in the total scoring.

In the Norwegian lifestyle index, the diet component was weighted equally as the other four lifestyle components, given the strong association with risk of adverse health outcomes (17). These included body weight, physical activity, tobacco use, and alcoholic beverages. Body weight was operationalized by BMI defined by the distinct BMI categories by WHO (15). The national recommendation regarding physical activity was updated in 2022 (16). The advice is to engage in regular physical activity at least in 150–300 min per week with moderate intensity and 75–150 min per week in vigorous intensity, or a combination of these, and to reduce time being sedentary, which is similar to the WHO recommendations (15, 16). Tobacco use was also included as an independent component. The 2018 WCRF/AICR-score, for instance, did not include tobacco in their index, but emphasized the importance of this component in the light of health issues. The final component of the healthy lifestyle index was alcohol. A low intake of alcohol is probably not an important risk factor for chronic diseases. Some studies have suggested that a moderate intake equals to two units per day for men and one unit per day for women (8, 13). However, the intake-response relationship is not precisely known. We choose a small amount, equal to 30 g of pure ethanol per week, which was accepted to achieve half score (0.5 points).

Low adherence to the diet index and high adherence to the four lifestyle components were predominantly in the national survey, Norkost3, indicating a great potential for improvements in adherence to the dietary recommendations. A wide range of adherence to each of the recommendations in the diet index were observed. Low adherences to several of the main components characterizing a healthy dietary pattern were observed, such as plant-based foods (e.g. fruits, vegetables, nuts, whole grains), fish, meat and foods rich in sugars and fat (9, 26). These components are associated with risk of chronic diseases such as cancer, cardiovascular diseases, diabetes type 2 and bone health, all with high prevalence in the Norwegian population (9, 26). In particular, the highest-ranked dietary risk factors for the burden of chronic diseases in the Nordic and Baltic countries are low intakes of whole grains and fruits, and high intakes of processed meat and red meat (26). Thus, there is a need for interventions to improve adherence to the diet components shown to have low adherence in the national survey.

The data from Norkost3 is based on two separate 24 h-recalls for each participant, which increases the quality of the diet assessment (27). However, a limitation in this study is the slightly overestimation of fish and meat intakes explained by the registration of fish and meat as raw or not prepared items in the Norkost3 database (e.g. about 2/3), and thus resulting slightly in higher weights in grams as compared with prepared foods. The recommended intakes in grams of fish and meat included in the Norwegian food index is based on prepared foods. We therefore performed a recalculation for fish and meat intakes. We considered that 1/3 of the food recorded in KBS is prepared. Estimation of preparation for the other part (i.e. 2/3) was conducted by multiplying with a correction factor accounting for the decreased weight due to the preparation process (28) for each food group (i.e. 0.66 for meat and 0.76 for fish), and then adding the other 1/3 part with prepared food. For fish, percentage of participants with high adherence to the recommendation decreased from 43 to 41%, whereas participants with intermediate and low adherences increased from 8 to 9% and from 48 to 50%, respectively. Moreover, percentage of participants with high and intermediate adherences to the recommendation of meat increased from 27 to 31% and 17 to 23%, respectively, and participants with low adherence decreased from 57 to 47%. By this, the highest impact of the correction into prepared food was seen for low adherence to the recommendation of meat.

Focusing on the recommendations with low adherence may be beneficial for interventions aiming at improving individual or public health. In the sensitivity analyses, we tested the contribution of each dietary components to the overall individual diet index. The plant-based components showed to have the highest potential in contributing to the adherence to the index.

A major strength of this article is the use of quantitative cut-off values for most of the components included in the final index, based on the specific recommendations and sub-recommendations in the Norwegian FBDGs. In cases where quantitative values were missing or lacking, the qualitative recommendations were translated into quantitative cut-off values as described in Henriksen et al. (12).

## Conclusion

In this article, we have developed a diet and a lifestyle index that assess adherence to the national guidelines in Norway, which can be used to predict several health outcomes. The indices reflect a healthy lifestyle pattern and there is a large potential in improving public health by focusing on the specific components that contribute to reduce overall scoring. Future updates in the guidelines can easily be implemented in the indices and they may be applicable in other countries with comparable national guidelines.

## References

[1] Dietary Guidelines Advisory Committee. Scientific report of the 2020 Dietary Guidelines Advisory Committee: Advisory report to the Secretary of Agriculture and the Secretary of Health and Human Services. Washington, DC: U.S. Department of Agriculture, Agricultural Research Service; 2020.

[2] Hu FB. Dietary pattern analysis: a new direction in nutritional epidemiology. Curr Opin Lipidol 2002; 13(1): 3–9. doi: 10.1097/00041433-200202000-00002 11790957

[3] Burggraf C, Teuber R, Brosig S, Meier T. Review of a priori dietary quality indices in relation to their construction criteria. Nutr Rev 2018; 76(10): 747–64. doi: 10.1093/nutrit/nuy027 30053192 PMC6130981

[4] Trichopoulou A, Costacou T, Bamia C, Trichopoulos D. Adherence to a Mediterranean diet and survival in a Greek population. N Engl J Med 2003; 348(26): 2599–608. doi: 10.1056/NEJMoa025039 12826634

[5] Olsen A, Egeberg R, Halkjaer J, Christensen J, Overvad K, Tjonneland A. Healthy aspects of the Nordic diet are related to lower total mortality. J Nutr 2011; 141(4): 639–44. doi: 10.3945/jn.110.131375 21346102

[6] Guenther PM, Casavale KO, Reedy J, Kirkpatrick SI, Hiza HA, Kuczynski KJ, et al. Update of the Healthy Eating Index: HEI-2010. J Acad Nutr Diet 2013; 113(4): 569–80. doi: 10.1016/j.jand.2012.12.016 23415502 PMC3810369

[7] Hu EA, Steffen LM, Coresh J, Appel LJ, Rebholz CM. Adherence to the Healthy Eating Index-2015 and other dietary patterns may reduce risk of cardiovascular disease, cardiovascular mortality, and all-cause mortality. J Nutr 2020; 150(2): 312–21. doi: 10.1093/jn/nxz218 31529069 PMC7373820

[8] Shams-White MM, Brockton NT, Mitrou P, Romaguera D, Brown S, Bender A, et al. Operationalizing the 2018 World Cancer Research Fund/American Institute for Cancer Research (WCRF/AICR) Cancer Prevention Recommendations: a standardized scoring system. Nutrients 2019; 11(7): 312–21. doi: 10.3390/nu11071572 31336836 PMC6682977

[9] Kostråd for å fremme folkehelsen og forebygge kroniske sykdommer: metodologi og vitenskapelig kunnskapsgrunnlag. Oslo: Nasjonalt råd for ernæring, Helsedirektoratet; 2011. 353 s, ill. p.

[10] Norwegian Directorate of Health. Available from: https://www.helsenorge.no/kosthold-og-ernaring/sma-grep-for-et-sunt-kosthold/dagens-maltider/#tallerkenmodellen [cited 01 Jan 2021].

[11] Henriksen HB, Berntsen S, Paur I, Zucknick M, Skjetne AJ, Bøhn SK, et al. Validation of two short questionnaires assessing physical activity in colorectal cancer patients. BMC Sports Sci Med Rehabil 2018; 10: 8. doi: 10.1186/s13102-018-0096-2 29854408 PMC5975662

[12] Henriksen HB, Carlsen MH, Paur I, Berntsen S, Bohn SK, Skjetne AJ, et al. Relative validity of a short food frequency questionnaire assessing adherence to the Norwegian dietary guidelines among colorectal cancer patients. Food Nutr Res 2018; 62: 1306. doi: 10.29219/fnr.v62.1306 PMC5846207 29545734

[13] World Cancer Research Fund/American Institute for Cancer Research. Continuous update project expert report 2018. Diet, nutrition and physical activity. World Cancer Research Fund/American Institute for Cancer Research, London, UK; 2018.

[14] Folkehelseinstituttet. Helsetilstanden i Norge 2018. [Public Health in Norway 2018] Norwegian Institute of Public Health, Oslo, Norway; 2018.

[15] WHO. WHO-physical activiy: World Health Organization (WHO); 2020. Available from: https://www.who.int/news-room/fact-sheets/detail/physical-activity [cited 29 Sep 2022].

[16] Helsedirektoratet. Fysisk aktivitet i forebygging og behandling: Norwegian Directorate of Health. 2019. Available from: https://www.helsedirektoratet.no/faglige-rad/fysisk-aktivitet-i-forebygging-og-behandling [cited 29 Sep 2022].

[17] Murray CJL, Aravkin AY, Zheng P, Abbafati C, Abbas KM, Abbasi-Kangevari M, et al. Global burden of 87 risk factors in 204 countries and territories, 1990–2019: a systematic analysis for the Global Burden of Disease Study 2019. Lancet. 2020; 396(10258): 1223–49. doi: 10.1016/S0140-6736(20)30752-2 33069327 PMC7566194

[18] Totland TH. Norkost 3: en landsomfattende kostholdsundersøkelse blant menn og kvinner i Norge i alderen 18–70 år, 2010–11. Oslo: Helsedirektoratet; 2012, 67 s. 30 cm p.

[19] Haines PS, Siega-Riz AM, Popkin BM. The Diet Quality Index revised: a measurement instrument for populations. J Am Diet Assoc 1999; 99(6): 697–704. doi: 10.1016/s0002-8223(99)00168-6 10361532

[20] Patterson RE, Haines PS, Popkin BM. Diet quality index: capturing a multidimensional behavior. J Am Diet Assoc 1994; 94(1): 57–64. doi: 10.1016/0002-8223(94)92042-7 8270756

[21] Soltani S, Arablou T, Jayedi A, Salehi-Abargouei A. Adherence to the dietary approaches to stop hypertension (DASH) diet in relation to all-cause and cause-specific mortality: a systematic review and dose-response meta-analysis of prospective cohort studies. Nutr J 2020; 19(1): 37. doi: 10.1186/s12937-020-00554-8 32321528 PMC7178992

[22] Herforth AW, Wiesmann D, Martínez-Steele E, Andrade G, Monteiro CA. Introducing a suite of low-burden diet quality indicators that reflect healthy diet patterns at population level. Curr Dev Nutr 2020; 4(12): nzaa168. doi: 10.1093/cdn/nzaa168 33344879 PMC7723758

[23] Estruch R, Ros E, Salas-Salvadó J, Covas M-I, Corella D, Arós F, et al. Primary prevention of cardiovascular disease with a Mediterranean diet supplemented with extra-virgin olive oil or nuts. N Engl J Med 2018; 378(25): e34. doi: 10.1056/NEJMoa1800389 29897866

[24] United Nations Development Programme. Sustainable Development Goals 2022. Available from: https://www.undp.org/sustainable-development-goals?utm_source=EN&utm_medium=GSR&utm_content=US_UNDP_PaidSearch_Brand_English&utm_campaign=CENTRAL&c_src=CENTRAL&c_src2=GSR&gclid=Cj0KCQjwxtSSBhDYARIsAEn0thQwBZPmQDdZF3SaofVT7P48nz3WyqBKHMPDNorKw71qxHthzOzv3eoaAs37EALw_wcB [cited 29 Sep 2022].

[25] Nasjonalt råd for ernæring: Bærekraftig kosthold-vurdering av de norske kostrådene i et bærekraftsperspektiv (Sustainable diet). Health NDo; 2017 Nov 2017. Report No.: IS-2678 Contract No.: IS-2678. Norwegian Directorate of Health, Oslo, Norway.

[26] Blomhoff RAR, Arnesen EK, Christensen JJ, Eneroth H, Erkkola M, Gudanaviciene I, et al.. Nordic nutrition recommendations. Copenhagen: Nordic Council of Ministers; 2023.

[27] Andersen LF, Berstad P, Bukhvalova BA, Carlsen MH, Dahl LJ, Goksøyr A, et al. Benefit and risk assessment of fish in the Norwegian diet – Scientific Opinion of the Steering Committee of the Norwegian Scientific Committee for Food and Environment. Benefit and risk assessment of fish in the Norwegian diet – Scientific Opinion of the Steering Committee of the Norwegian Scientific Committee for Food and Environment. Norwegian Institute of Public health, Oslo, Norway; 2022.

[28] Dalane JØ, Bergvatn TAM, Kielland E, Carlsen MH. Mål, vekt og porsjonsstørrelser for matvarer = Weights, measures and portion sizes for foods. Oslo: Mattilsynet Universitetet i Oslo Helsedirektoratet; 2015.

[29] Solans M, Chan DSM, Mitrou P, Norat T, Romaguera D. A systematic review and meta-analysis of the 2007 WCRF/AICR score in relation to cancer-related health outcomes. Ann Oncol 2020; 31(3): 352–68. doi: 10.1016/j.annonc.2020.01.001 32067678

