# Supplementary material for: Development of the Norwegian diet index and the Norwegian lifestyle index and evaluation in a national survey
Source: Food Nutr Res. 2023 Sep 29;67:10.29219/fnr.v67.9217. doi: 10.29219/fnr.v67.9217 (PMC10552792; doi:10.29219/fnr.v67.9217)
Supplement: Supplementary file 1 [file FNR-67-9217-s001.docx]

**Supplementary table 1.** Mean (95 % confidence interval) dietary intake of energy and food groups included in the Norwegian diet index, and comparison between genders in Norkost3.

| **Food group** | **Total (n=1787)** | **Men (n=862)** | **Women (n=925)** | **Mean difference  between genders** | |
| --- | --- | --- | --- | --- | --- |
| Energy, MJ/d | 9.4 (9.3, 9.6) | 10.9 (10.7, 11.2) | 8.0 (7.9, 8.2) | 2.9 (2.6, 3.2) | |
| Fruit and berries^1^, g/d | 192 (185, 199) | 179 (168, 189) | 205 (195, 214) | -26 (-40, -12) | |
| Vegetables^2^, g/d | 141 (136, 146) | 137 (130, 144) | 144 (138, 151) | -7 (-17, 2) | |
| Whole grains^3^, g/d | 54 (52, 56) | 61 (58, 64) | 48 (46, 50) | 13 (9, 17) | |
| Unsalted nuts, g/d | 4 (3, 5) | 4 (3, 5) | 4 (3, 5) | -1 (-2, 1) | |
| Fish (fatty and lean fish), g/d | 62 (58, 66) | 74 (67, 81) | 52 (47, 56) | 23 (15, 31) | |
| Low-fat dairy products^4^, g/d | 269 (255, 283) | 337 (313, 360) | 206 (192, 221) | 130 (103, 158) | |
| Margarine and oils, g/d | 15 (14, 15) | 18 (17, 20) | 11 (10, 12) | 8 (6, 9) | |
| Red meat^5^, g/d | 108 (103, 112) | 135 (127, 143) | 82 (78, 87) | 53 (43, 62) | |
| Processed meat^6^, g/d | 74 (70, 77) | 92 (86, 98) | 57 (53, 61) | 35 (28, 42) | |
| Foods rich in sugar and fat^7^, g/d | 82 (78, 85) | 87 (81, 93) | 77 (72, 81) | 10 (3, 18) | |
| Drinks with added sugar, g/d | 129 (116, 141) | 171 (151, 192) | 89 (75, 102) | 82 (58, 107) | |
|  |  |  |  |  | |
|  |  |  |  |  | |
| ^1^ Include max one glass (200 grams) of juice as one portion of fruit (100 grams), not jam  ^2^ Not included legumes and potatoes | | | | |  |
| ^3^ Intake of whole grains was calculated using a whole grain factor (with the assumption that bread contains 60 % flour and boiled rice/pasta contains 30 % cereal): | | | | |  |
| Bread with 0–25% wholemeal flour: (60*0)/10,000 = 0 | | | | |  |
| Bread with 25–50% wholemeal flour: (60*25)/10,000 = 0.15 | | | | |  |
| Bread with 50–75% wholemeal flour: (60*50)/10,000 = 0.30 | | | | |  |
| Bread with 75–100% wholemeal flour: (60*75)/10,000 = 0.45 | | | | |  |
| Whole grain crisp bread = 1 | | | | |  |
| Sweetened cereals = 0.25 | | | | |  |
| Unsweetened cereals = 0.75 | | | | |  |
| Brown rice = 0.30 | | | | |  |
| Whole grain pasta = 0.30 | | | | |  |
| ^4^ Includes lean milk with less than 1.5% fat, dairy products (not cheese and milk) containing more than 20 % fat and/or energy content more than 950-1150 kJ and cheese containing less than 17% fat, cheese labelled with light/fat reduced or containing energy less than 950-1150 kJ  ^5^ Includes non-processed and processed beef, pork, lamb and goat  ^6^ Includes all forms of processed meat  ^7^ Includes cakes, dessert, ice-cream, candy and snacks | | | | |  |
